# Fine motor skills of the hands in Polish and Czech female senior citizens from different backgrounds

**DOI:** 10.1007/s40520-014-0299-7

**Published:** 2014-12-18

**Authors:** Anna Skrzek, Miroslava Přidalová, Anna Sebastjan, Dominika Harásková, Jaroslaw Fugiel, Zofia Ignasiak, Teresa Slawinska, Krystyna Rozek

**Affiliations:** 1 Faculty of Physiotherapy, University School of Physical Education in Wroclaw, Wroclaw, Poland; 2 Faculty of Physical Education, University School of Physical Education in Wroclaw, al. Paderewskiego 35, 51-612 Wroclaw, Poland; 3 Faculty of Physical Culture, Palacký University in Olomouc, Olomouc, Czech Republic

**Keywords:** Fine motor skills, Aging, Physical activity, University of the Third Age

## Abstract

The aim of the present study was an in-depth analysis of fine motor skills of the hands in elderly women from different socio-cultural backgrounds. The research also included analysis of the associations of age with the variables assessing right- and left-hand motor skills and its effect on hand performance asymmetry. The study examined 486 women over the age of 60. The study measured dominant and non-dominant hand performance using the motor performance series test battery (aiming, line tracking, inserting pins, tapping) from the Vienna test system. The best results in the tests assessing coordinated hand movements were achieved by the group of elderly women attending a University of the Third Age in Poland. This may be the result of a larger variety of physical activity programs offered at this type of institution. However, due to the cross-sectional design of the study, additional research of a longitudinal nature needs to be performed using the same sample of individuals to draw any definitive conclusions. Additionally, a decrease in the differences between dominant and non-dominant hand function with age was observed.

## Introduction

The diverse and multi-faceted physiological changes associated with senescence are marked by a gradual deterioration of function in elderly adults. One significant component of the aging process is a decline in motor coordination, leading to problems with walking, an increased fall risk, and, eventually, a lack of independence [1, 2]. The effects of aging may also impair smaller muscle movements such as those performed by the hand, leading to severe problems in performing even the most basic activities of daily living.

Throughout life, our hands go through many age-related anatomical and physiological changes influenced by numerous internal and external factors [3, 4]. Hand function may be impaired by the degenerative loss of skeletal muscle mass (sarcopenia), causing a decline in both strength and endurance [5–7]. Kalisch et al. [3] determined that normal hand function requires adequate muscle strength, where a grip strength of 9 kg (20 lb) is considered to be the minimum to perform activities of daily living [8]. Biological aging also induces deleterious effects on hand function through impaired eye-hand coordination, where the functioning of the eyes and hands can lead to degraded fine motor skill performance. All of the above factors determine whether an elderly individual is able to adequately perform activities of daily living and function independently [9].

A decrease in hand function may also result from changes in the peripheral nervous system, such as a reduction in nerve conduction velocity, sensory perception, or the excitation–contraction coupling of motor units [10, 11]. It has been suggested that a lower tolerance to muscle fatigue in the elderly is associated with changes in both muscle and the central nervous system [6]. In addition, impaired sensory perception is considered to be responsible for reduced sensorimotor performance [12]. Altogether, the complex age-related changes in the physiology of the human brain and entire central nervous system are manifested by a decrease in perception and cognitive ability, slowed motor activity, and a loss of various motor skills [13]. Furthermore, the ability to repeat movements at a high frequency is largely dependent on how the central nervous system regulates antagonistic muscle groups and the mechanism of muscle activation and inhibition. These aspects are also influenced by the aging process, where again a quicker onset of fatigue has been observed in the elderly. Even activities requiring relatively little strength have seen the elderly use a proportionately greater number of motor units than younger individuals [14].

A number of authors have indicated that impaired hand motor function places significant limitations on performing basic life tasks [4, 5, 9, 15]. Besides an objective assessment of changes in motor skill performance, it is important for researchers to better examine the influence of various environmental factors on the aging process, such as the influence of physical activity [16, 17] and whether a sedentary lifestyle may contribute to impaired hand function [5, 18].

It is believed that factors such as a lack of physical activity and feelings of isolation significantly accelerate the aging process. Today’s approach to the issue of old age has recognized the need to socially activate the elderly by providing them with opportunities to expand their social circle, fulfill their needs of self-development, and allow them to further education and acquire new skills. To meet these challenges, Universities of the Third Age (U3A) are heavily involved in promoting an active lifestyle for seniors. These educational institutions provide educational opportunities and increase the social inclusion of older adults (individual and community-wise), take advantage of their potential in terms of knowledge, skills, and experience, as well as encourage physical fitness and an active approach to overall health. Many Universities of the Third Age follow the French model, which is based on close collaboration with academic institutions. It involves high-level teaching activities and scientific research involving a wide range of programs such as lectures, seminars, interest groups, art workshops, recreational activities, exercise classes, physical therapy, cultural events, tourist trips, and many others. Involvement in such educational programs and the various activities proposed by Universities of the Third Age can help reduce the negative effects of aging such as decreased hand function, which, combined with preventive gerontological measures, may help reduce the incidence of disability and dependence on outside forms of care.

### Study purpose

The aim of the study was to therefore analyze the effects of age on the fine motor skills of the hand in a group of elderly women from different socio-cultural backgrounds (individuals attending a local University of the Third Age and peers not involved in any other similar type of educational/physical activity program). This included evaluating the association of age with changes in the dominant and non-dominant hand and their influence on the asymmetry of hand function. Although the literature has shown a strong relationship between hand dominance and hand performance in young, healthy individuals [19, 20], mixed results have been published regarding the age-related changes associated with right- and left-handedness and the asymmetry of hand function with aging [3, 19, 20].

## Materials and methods

The study examined 486 women over the age of 60 recruited from a variety of backgrounds. The first group (U3A-P) consisted of 90 women attending a University of the Third Age operating in the cities of Wroclaw and Jelenia Gora, located in the southwest of Poland. The second group (U3A-Cz) included 53 women attending a University of the Third Age in the city of Olomouc, Czech Republic. The third group (S) was composed of independent female senior citizens from the southwest region of Poland that voluntarily participated in the study and were not involved in any organized physical activity program. Each participant met the following inclusion criteria:

- age over 60 years
- right-handedness
- functional independence and good, overall health
- no upper extremity disorders
- written consent to participate in the study

Descriptive statistics of the participants’ age, body mass and height, and body mass index (BMI) are provided in Table 1. No statistically significant differences were found between the three groups for age and mass and height variables.

**Table 1:** Descriptive characteristics by group for age, body mass and height, and BMI

Variables	U3A-P *n* = 90		U3A-Cz *n* = 53		S *n* = 343	
	$\bar{x}$	SD	$\bar{x}$	SD	$\bar{x}$	SD
Age (years)	64.06	2.82	63.73	2.68	64.40	3.24
Body height (cm)	160.27	4.93	162.43	5.99	158.71	5.52
Body mass (kg)	70.81	12.24	73.17	13.55	71.68	11.72
BMI (kg/m^2^)	27.55	4.54	27.79	5.27	28.49	4.68

All of the participants were informed of the study’s purpose and procedure and the conditions for participation. The part of the study performed in Poland was conducted under the auspices of the Ministry of Science and Higher Education (no. N404 075337) at the Biokinetics Laboratory headed by the Department of Biostructure of the University School of Physical Education in Wroclaw, Poland. Research performed in the Czech Republic was conducted as part of a project titled “Physical activity and inactivity of inhabitants of the Czech Republic in context of behavioral changes” (MSM 6198959221) at the Palacký University of Olomouc. Both projects received the approval of their local ethics committee.

The study measured hand performance (fine motor skills) using the Motor Performance Series (MLS) from the Vienna Test System (S3 short form as adapted by Vassella). This test battery was developed by K. J. Schoppe, which is based on the work of W. Hamstel (1980) on specific motor skills using factor analysis. The test battery uses a device in the form of a work panel with various contact surfaces where a stylus is used to perform static and dynamic tasks. For the purposes of this study, six motor skills were assessed, aiming (movement accuracy), hand shake (tremor), arm-hand movement precision, hand-finger dexterity, arm-hand movement speed, wrist-finger speed, using the MLS’ Aiming, line tracking, inserting pins, and tapping tests. The tests, performed by the participants with both the right (dominant) and left (non-dominant) hands, are explained in greater detail below:

- Aiming involves using the stylus to touch 20 holes (5 mm diameter placed 4 mm apart) as quickly as possible without touching the baseplate (scored by the time needed to finish the task) to measure small-range movements,
- Line tracking is performed by inserting the stylus in a groove and outlining its shape without touching the sides or bottom of the baseplate (scored by the number of times the stylus touched the surface, or errors) to measure arm-hand movement precision,
- Inserting pins requires the participant to pick up pins found in a container 30 cm from the work panel and place them one by one into 25 holes (scored by the time needed to finish the task) to measure hand and finger dexterity,
- Tapping is a task where the stylus is used to strike a special 40 × 40 mm square surface as many times as possible within 32 s (scored by the number of accurate hits) to measure wrist-finger movement speed.

### Statistical analysis

Normality of the data was assessed using the Shapiro–Wilk test, finding all of the parameters to have normal distribution. Descriptive statistics were then calculated, including means, standard deviations, and coefficients of variation. Comparison of the mean values between the groups was performed using analysis of variance (ANOVA). Post hoc analysis was performed with the least significant difference test (LSD). Simple linear regression was used to determine the association of the analyzed parameters with age. Statistical significance was set at *p* ≤ 0.05 throughout. Statistical analyses were performed using Statistica 9.1 software.

## Results

Analysis of the differences in the performance of tests measuring the fine motor skills of the dominant and non-dominant hand found statistically significantly better test results obtained with the dominant hand. This was observed in all the groups of elderly women (Tables 2, 3). For the dominant hand, the worst results were obtained by the seniors (S) not involved in any organized physical activity program when compared with U3A women; the differences were statistically significant for all of the analyzed mean test results except for the time needed to finish the Aiming test. No statistically significant intergroup differences were found when comparing the elderly U3A students from Poland and the Czech Republic, where the tests results of both groups’ fine motor skills were similar (Tables 2, 3).

**Table 2:** Descriptive characteristics by group for the hand performance tests

Test parameter	UE	U3A-P *n* = 90			U3A-Cz *n* = 53			*S* *n* = 343		
		$\bar{x}$	SD	CV	$\bar{x}$	SD	CV	$\bar{x}$	SD	CV
Aiming (s)	R	9.57	2.24	23.39	9.86	1.83	18.51	10.14	2.20	21.73
	L	10.85	2.35	21.64	11.93	1.96	16.44	11.01	2.62	23.78
Line tracking (*n*)	R	23.54	8.13	34.51	21.04	11.94	56.77	26.02	9.48	36.42
	L	30.29	9.93	32.78	29.21	10.81	37.02	33.24	10.77	32.39
Inserting pins (s)	R	42.99	4.48	10.43	42.79	5.58	13.04	44.70	5.62	12.57
	L	48.20	5.29	10.97	47.13	5.14	10.91	49.96	6.65	13.30
Tapping (*n*)	R	186.01	19.16	10.30	186.94	17.71	9.47	180.14	20.37	11.31
	L	170.02	19.19	11.28	170.49	21.95	12.87	162.66	18.86	11.59

*UE* upper extremity, *R* right (dominant), *L* left (non-dominant), $\bar{x}$ mean, *SD* standard deviation, *CV* coefficient of variation

**Table 3:** Assessment of differences in the hand performance tests for the right (dominant) and left (non-dominant) extremity by group including between-group differences

Test parameter	U3A-P	U3A-Cz	*S*	R			L		
	R/L			U3A-P U3A-Cz	S/U3A-P	S/U3A-Cz	U3A-P U3A-Cz	S/U3A-P	S/U3A-Cz
Aiming	**0.0000**	**0.0000**	**0.0000**	0.4694	**0.0413**	0.4293	**0.0079**	0.5586	**0.0081**
Line tracking	**0.0000**	**0.0000**	**0.0000**	0.1521	**0.0387**	**0.0009**	0.5365	**0.0138**	**0.0070**
Inserting pins	**0.0000**	**0.0000**	**0.0000**	0.8420	**0.0142**	**0.0276**	0.2939	**0.0115**	**0.0011**
Tapping	**0.0000**	**0.0000**	**0.0000**	0.7834	**0.0115**	**0.0188**	0.8902	**0.0016**	**0.0069**

Statistically significant differences at *p* ≤ 0.05 marked in bold

*R* right (dominant) extremity, *L* left (non-dominant) extremity

**Fig. 1:**
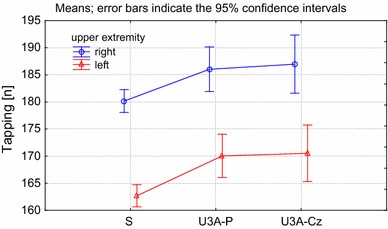
Between-group differences in the mean number of accurate hits in the tapping test

A similar situation was observed in the case of the non-dominant hand; in most tests the lowest scores were obtained by the seniors not involved with the U3A. The U3A students from Poland and the Czech Republic achieved similar results except for the time to complete the Aiming test, in which the Polish seniors performed statistically significantly better than their Czech peers (Tables 2, 3). An example of the differences between the seniors not involved in a physical activity program and those attending a U3A are presented in the Tapping test (Fig. 1).

**Fig. 2:**
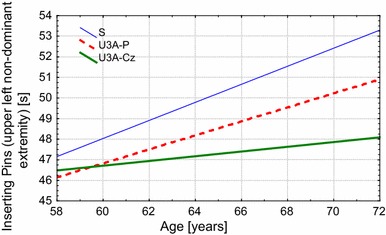
Differences in hand performance changes with age in the inserting pins test

Analysis of the changes in fine motor skill performance among the surveyed women showed a deterioration in the results of all tests with age mainly in the group of less physically active seniors (group S) (Table 4). In addition, group S also achieved significantly poorer results with age than their U3A peers. These changes applied to all of the evaluated parameters for both upper extremities. In the group of seniors (S) not participating in an organized physical activity program, the decrease in most of the test results with age was statistically significant (Table 4). An example of the changes associated with age, statistically significant in all groups, is presented with the results of the Inserting Pins test (Fig. 2).

**Table 4:** Regression coefficients of the analyzed parameters as a function of age

Test parameter	UE	Group	Intercept				Effect of age			
			*b*_0_	Std. error *b*_0_	*t*	*p*	*b*_age_	Std. error *b*_age_	*t*	*R*
Aiming	R	U3A-P	−0.105	5.320	−0.02	0.9843	0.151	0.083	1.82	0.0721
		U3A-Cz	7.154	6.069	1.18	0.2440	0.043	0.095	0.45	0.6570
		S	0.685	2.318	0.30	0.7678	0.147	0.036	4.08	**0.0001**
	L	U3A-P	16.439	5.654	2.91	**0.0046**	−0.087	0.088	−0.99	0.3252
		U3A-Cz	3.761	6.431	0.58	0.5612	0.128	0.101	1.27	0.2092
		S	4.254	2.799	1.52	0.1295	0.105	0.043	2.42	**0.0161**
Line tracking	R	U3A-P	37.769	19.617	1.93	0.0574	−0.222	0.306	−0.73	0.4699
		U3A-Cz	−86.809	36.792	−2.36	**0.0222**	1.692	0.577	2.93	**0.0050**
		S	4.075	10.145	0.40	0.6882	0.341	0.157	2.17	**0.0310**
	L	U3A-P	35.924	24.036	1.49	0.1386	−0.088	0.375	−0.23	0.8150
		U3A-Cz	−27.660	35.114	−0.79	0.4345	0.892	0.551	1.62	0.1112
		S	12.606	11.553	1.09	0.2760	0.320	0.179	1.79	0.0746
Inserting pins	R	U3A-P	20.607	10.589	1.95	0.0549	0.350	0.165	2.12	**0.0371**
		U3A-Cz	−10.862	16.996	−0.64	0.5256	0.842	0.266	3.16	**0.0027**
		S	22.934	5.939	3.86	**0.0001**	0.338	0.092	3.67	**0.0003**
	L	U3A-P	26.419	12.596	2.10	**0.0388**	0.340	0.196	1.73	0.0870
		U3A-Cz	39.819	17.102	2.33	**0.0239**	0.115	0.268	0.43	0.6704
		S	21.718	6.998	3.10	**0.0021**	0.438	0.109	4.04	**0.0001**
Tapping	R	U3A-P	152.978	46.256	3.31	**0.0014**	0.516	0.721	0.71	0.4766
		U3A-Cz	287.629	57.254	5.02	**0.0000**	−1.580	0.898	−1.76	0.0844
		S	225.903	21.819	10.35	**0.0000**	−0.711	0.338	−2.10	**0.0365**
	L	U3A-P	129.153	46.252	2.79	**0.0064**	0.638	0.721	0.88	0.3789
		U3A-Cz	223.636	72.710	3.08	**0.0034**	−0.834	1.140	−0.73	0.4678
		S	206.070	20.193	10.20	**0.0000**	−0.674	0.313	−2.15	**0.0321**

Statistically significant differences at *p* ≤ 0.05 marked in bold

*UE* upper extremity, *R* right (dominant), *L* left (non-dominant)

**Fig. 3:**
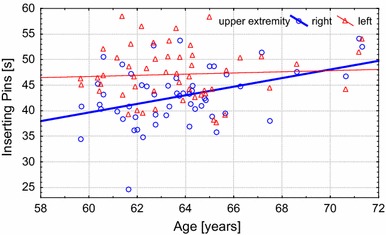
Changes with age in the inserting pins test (total time) between the dominant and non-dominant extremities in the U3A-Cz group

**Fig. 4:**
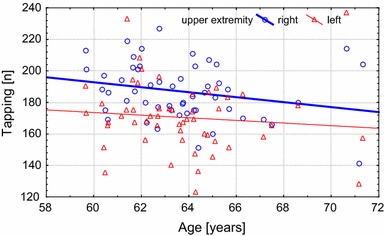
Changes with age in the tapping test (number of accurate hits) between the dominant and non-dominant extremities in the U3A-Cz group

The results show that more significant age-related changes occur with the right (dominant) hand. In addition, the differences between the dominant and non-dominant hand decreased with age (Table 4). An example of the changes in hand performance asymmetry can be observed in the results of the Inserting Pins (Fig. 3) and tapping (Fig. 4) tests between the dominant and non-dominant extremity in the U3A-Cz group.

## Discussion

A temporary or permanent impairment in performing fine motor skills with the upper extremities is seen as one of the most troublesome aspects of aging. It may severely affect activities of daily living, self-management, and self-care, leading to poorer quality of life and reduced functional independence in the elderly [21]. The dynamics of these changes are highly variable, depending on age, sex, and many other internal and external factors [22].

As expected, a decrease in most of the analyzed hand performance measures was found with age. In most of the tests, increasing age was associated with a decreasing number of properly completed tasks and increased test-taking time.

These changes, indicating a deterioration of fine motor skills, were statistically significant in most cases. This may be attributed to the fact that a decrease in muscle mass has been strongly correlated with coordination, which affects the proper functioning of the upper extremities in terms of performing small, precise movements [23]. One of the most important time periods associated with hand dexterity appears to be after 60 years of age [12]. A number of researchers have observed that the mechanisms associated with coordination begin to slow down and become increasingly unharmonized around this age, where movements become increasingly disturbed and therefore less accurate and smooth [5, 24, 25]. However, due to the cross-sectional design of the study, additional research of a longitudinal nature needs to be performed using the same sample of individuals to draw any definitive conclusions.

Mattay et al. [13] or Cabeza [26] used MRI to detect the decreases in motor skill performance as a function of aging by observing the structural and neurochemical changes in the aging brain. The findings of their studies indicate that the elderly engage additional cortical and sub-cortical areas of the brain to perform even simple tasks. This may result from the use of various compensatory mechanisms in the aging brain, such as the reorganization and redistribution of functional processes. Such results may indicate that brain activity during movement in older adults is clearly different from than in young individuals and may be associated with nerve cell loss in the brain and aspects concerned with metabolism and circulation.

It is widely accepted that tapping is an activity that can accurately check the performance of the central nervous system and the functioning of effectors, which has been confirmed for example by Głowacki et al. [14]. Christianson [27] showed significant differences in the results of a tapping test between individuals aged 25–39 and 55–70 years. Cousins et al. [28] and Bodwell et al. [29] also compared a younger and older sample, finding older adults had slower reaction times and longer movement duration. In the present study, the results of the tapping test confirmed this observation. The number of accurate hits decreased with age, particularly in the group of physically inactive seniors where the decrease was statistically significant.

Although it has been repeatedly confirmed that the dominant hand is more dexterous than the non-dominant hand in young individuals, little is still known about the possible changes in the asymmetry of hand performance with age. Studies available in the literature that have analyzed this issue include the works of Bishop et al. [30], Corey et al. [31], and Henkel et al. [20], all of whom noted a decrease in dominant hand preference with age. Kalisch et al. [3] studied the fine motor skills of the hands also using the MLS tests as in the present study on individuals aged 20–90 years, confirming a similar trend where preference for one side of the body decreased with age. However, the large range of ages and small study samples raises doubts about the results of the above studies.

In the present study, the results of the MLS tests determining the fine motor skills of the hands showed statistically significant differences between the right (dominant) and left (non-dominant) hand in all of the groups of women. However, regression analysis presented a statistically significant decrease in the MLS test results with age more for the right (dominant) than left (non-dominant) extremity. This confirms that the differences in the functioning of the dominant and non-dominant hand, which are easily observant in younger individuals, decrease with age with the difference statistically significant for the dominant hand. On this basis it can be concluded that the asymmetry of hand function decreases with age, where these age-related changes are greater for the dominant than non-dominant hand, and giving rise to the notion that handedness may gradually decrease in subsequent age groups. However, it should be stressed that only individuals who declared to be right-handed participated in the present study.

The primary purpose of the present study was to analyze the differences and the rate of age-related changes associated with the fine motor skill performance of elderly women from different socio-cultural backgrounds in terms of lifestyle and their involvement in physical exercise as well as educational and social activities. The women attending a University of the Third Age were found to be highly active in numerous aspects of life in comparison with the group of seniors not participating in any organized educational/physical activity program. It has been observed that elderly individuals attending a U3A were altogether an intellectually and physically active group as confirmed in studies on their motor activity and ways of spending leisure time [32, 33].

The phenomenon of an aging population is a worldwide trend. At present there are already a number of institutions supporting the social activation of the elderly. In Europe, the first and foremost example of such an initiative is the spread of Universities of the Third Age. They fulfill an important role in the lives of the elderly and aim to actively improve their quality of life. In the literature, however, there are few reports that have analyzed the effects of aging on the fine motor skills of the hands and an active lifestyle. One such study addressing this issue was by Kalisch et al. [3], who described the limitations of performing everyday activities due to a deterioration of motor skills related to a sedentary lifestyle. Their study cited the works of Dik et al. [16] and Weuve et al. [17], who documented a relationship between physical activity and the fine motor skills of the hands in an elderly sample. This issue was also cursorily examined in studies by Ranganathan et al. [5], Horowitz et al. [34], and Shechtman et al. [8], who analyzed differences in the aging process in regard to age, sex, cognitive function, and other environmental factors.

In light of the previously reported results and the findings of the present study it can be confirmed that there is a decrease in fine motor skill performance of the hands in elderly women with age. These observed changes were larger in magnitude for the dominant extremity, albeit the difference in hand performance between the dominant and non-dominant hand decreased with age. However, the most striking difference was seen in the functional capabilities of the hand between the groups of elderly women in regards to their background and participation in physical activity.

In the present study, all of the tests analyzing hand fine motor skills found that the low mean test values indicate poorer motor skills in the group of less active women not involved in a U3A. In all of the tests except for Aiming, statistically significant differences were found between the Polish and Czech students attending a U3A and their non-active peers from Poland for both the dominant and non-dominant limb. The data presented herein, gives rise to the need to conduct further comparative analysis of upper extremity motor function. Taking into consideration that life expectancy has steadily increased over time, particularly in developed countries, the issue of determining predictors of aging is an important issue. Preventive strategies aimed at limiting the adverse effects of the aging process on the upper extremities can improve the functional capabilities of elderly adults in performing activities of daily living and improve their independence and quality of life.

## Conclusions

1. The best results in the tests assessing coordinated hand movements were achieved by the group of elderly women attending a University of the Third Age in Poland. This may be the result of a larger variety of physical activity programs offered at this type of institution. However, due to the cross-sectional design of the study, additional research of a longitudinal nature needs to be performed using the same sample of individuals to draw any definitive conclusions.
2. Significant age-related changes were found in all of the groups only for the ‘inserting pins’ task with the dominant hand. In the other tests assessing fine motor skills of the hands, a statistically significant decline in performance with age was observed in the group of non-active seniors.
3. Additionally, a decrease in the differences between dominant and non-dominant hand function with age was observed. The decrease in asymmetry arises from a more dynamic decrease in the efficiency of the dominant hand.
4. On the basis of the results it can be acknowledged that the active lifestyle characteristic of the elderly women participating in the University of the Third Age program is associated with better upper extremity function in terms of fine motor skill performance.
